# Genetically engineered microorganisms for the detection of explosives’ residues

**DOI:** 10.3389/fmicb.2015.01175

**Published:** 2015-10-29

**Authors:** Benjamin Shemer, Noa Palevsky, Sharon Yagur-Kroll, Shimshon Belkin

**Affiliations:** Department of Plant and Environmental Sciences, The Alexander Silberman Institute of Life Sciences, The Hebrew University of JerusalemJerusalem, Israel

**Keywords:** explosives, landmines, microbial bioreporters, biosensors, bioluminescence, 2,4,6- trinitrotoluene, 2,4-dinitrotoluene

## Abstract

The manufacture and use of explosives throughout the past century has resulted in the extensive pollution of soils and groundwater, and the widespread interment of landmines imposes a major humanitarian risk and prevents civil development of large areas. As most current landmine detection technologies require actual presence at the surveyed areas, thus posing a significant risk to personnel, diverse research efforts are aimed at the development of remote detection solutions. One possible means proposed to fulfill this objective is the use of microbial bioreporters: genetically engineered microorganisms “tailored” to generate an optical signal in the presence of explosives’ vapors. The use of such sensor bacteria will allow to pinpoint the locations of explosive devices in a minefield. While no study has yet resulted in a commercially operational system, significant progress has been made in the design and construction of explosives-sensing bacterial strains. In this article we review the attempts to construct microbial bioreporters for the detection of explosives, and analyze the steps that need to be undertaken for this strategy to be applicable for landmine detection.

## Introduction

The extensive production and use of explosives for both civilian and military purposes throughout the past century has created diverse environmental problems, including the contamination of soil and groundwater with explosive residues. Evidence for the toxic and mutagenic effects of explosive contaminants, such as 2,4,6-trinitrotoluene (TNT), hexahydro-1,3,5-trinitro-1,3,5-triazine (RDX), and octahydro-1,3,5,7-tetranitro-1,3,5,7-tetrazocine (HMX), is well established (Berthe-Corti et al., 1998; Schäfer and Achazi, 1999; Frische, 2002; Rosen and Lotufo, 2007). The international Agency for Research on Cancer (IARC) lists 2,4-dinitrotoluene (2,4-DNT) and 2,6-dinitrotoluene (2,6-DNT) as possible carcinogens in humans (IARC, 1996), and has determined that evidence regarding the carcinogenic potential of TNT is still inadequate. Substantial evidence for the contamination of groundwater reservoirs with explosives has been found in proximity to explosive manufacturing facilities (Funk et al., 1993; Schmelling et al., 1997; Spain et al., 2000; Bernstein et al., 2008).

Current detection and quantification methods for trace explosives in soil and groundwater mostly rely on analytical devices such as gas or liquid chromatography coupled with mass spectrometry. Although highly accurate and extremely sensitive, such analytical methodologies depend upon expensive equipment that is restricted to specialized laboratories and requires a high degree of expertise. A potential complementary approach, which provides information also on the bioavailability and toxicity of the target compounds is based on the use of live cell sensors (Belkin, 2003; Van der Meer and Belkin, 2010). Such bioreporters have also been proposed (Burlage, 2003; Garmendia et al., 2008; Yagur-Kroll et al., 2014) as a tool for the remote detection of buried landmines, out of which traces of explosives’ vapors have been demonstrated to leak and accumulate in the soil around them (Jenkins et al., 2001).

The most common explosive material present in both antipersonnel and antitank landmines is TNT, sometimes in combination with RDX (Jenkins et al., 2001; MacDonald et al., 2003). Two volatile impurities that accompany TNT are 1,3-dinitrobenzene (1,3-DNB) and – more prominently – 2,4-DNT. Although the latter accounts for less than 1% of the explosive material, its vapor pressure is much higher than that of TNT, resulting in higher concentrations of 2,4-DNT at ground level (MacDonald et al., 2003); this compound is therefore considered the most reliable landmine “signature” chemical (Jenkins et al., 2001).

## Microbial Bioreporters

Bioreporters are microbial strains genetically engineered to produce a dose-dependent quantifiable signal in response to the presence of pre-determined specific chemicals, groups of chemicals or stress factors. Numerous bioreporters have been described over the last two decades, mostly in the context of environmental monitoring, targeting either specific compounds such as heavy metals (Holmes et al., 1994; Corbisier et al., 1996; Belkin et al., 1997; Magrisso et al., 2008) or hydrocarbons (Applegate et al., 1998; Tecon et al., 2009), or global biological effects such as toxicity or genotoxicity (Vollmer et al., 1997; Biran et al., 2010). General and specific aspects of bacterial bioreporters’ construction and characterization have been described in numerous review articles over the last few years (Van der Meer and Jaspers, 2004; Marqués et al., 2006; Yagi, 2007; Van der Meer and Belkin, 2010; Choffnes et al., 2011; Eltzov and Marks, 2011; Roda et al., 2011; Su et al., 2011; Michelini et al., 2013; Cevenini et al., 2015) and will thus not be discussed in the present communication, which focuses only on microbial bioreporters tailored to sense and report upon the presence of trace explosives.

## Whole-Cell Bioreporters for the Detection of Explosives

### Bacteria

A list of microbial sensor strains previously reported to detect explosive-related chemicals is presented in **Table 1**.

**Table 1:** Reported attempts to construct microbial bioreporters for explosives’ detection.

Organism	Reporting element	Target analytes^(A)^	Reference
Unspecified bacterium	GFP	TNT	Burlage et al., 1999
*Vibrio fischeri*^(B)^	Intrinsic *lux* operon	TNT, 4A-DNT, 2A-DNT	Frische, 2002
*Escherichia coli*	GFP	TNT, L-lactate, serotonin	Looger et al., 2003^(C)^
*Dictyosphaerium chlorelloides*	Intrinsic chlorophyll A fluorescence	TNT	Altamirano et al., 2004
*Saccharomyces cerevisiae*	GFP	DNT	Radhika et al., 2007
*Pseudomonas putida*	*luxAB*, GFP	DNT	Garmendia et al., 2008
*Escherichia coli*	Flagellar motion	nitrite, nitrate	Kim et al., 2008
*Escherichia coli*	GFP	DNT	Lönneborg et al., 2012
*Escherichia coli*	GFP	DNT	Davidson et al., 2012
*Escherichia coli*	*GFPmut2, luxCDABE*	DNT, TNT	Yagur-Kroll et al., 2014
*Escherichia coli*	GFP	DNT, TNT, DNB	Tan et al., 2015

*^(A)^ TNT: 2,4,6-trinitrotoluene; DNT: 2,4-dinitrotoluene; 4A-DNT: 4-amino-2,6-dinitrotoluene; 2A-DNT: 2-amino-4,6-dinitrotoluene; DNB: dinitrobenzene; ^(B)^ Now *Aliivibrio fischeri*; ^(C)^ Results were contested by Reimer et al. (2014).*

**FIGURE 1:**
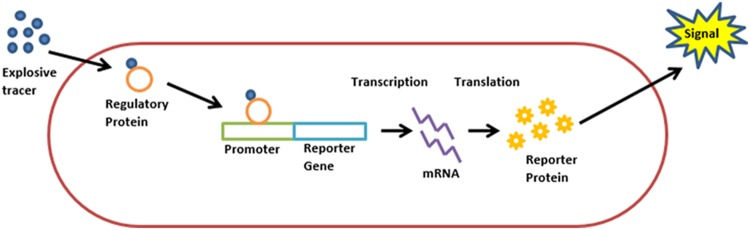
**Schematic description of a “lights on” bioreporter design.** A target analyte molecule enters the cell. The analyte, or its metabolite, is identified by a regulatory protein, which then activates the promoter attached to the reporter gene. Transcription is initiated, resulting in the synthesis of a reporter protein and the production of a measurable signal.

Burlage et al. (1999) were the first to suggest the use of recombinant bacteria as bioreporters for this purpose. The proposed scheme was simple: bacteria, genetically engineered to fluoresce upon exposure to TNT and 2,4-DNT (**Figure 1**), are sprayed on the area targeted for landmine clearance. The bacteria are then allowed to rest for two hours, in the course of which cells in the proximity of buried explosives will be exposed to TNT vapors, resulting in the activation of the reporter gene. By scanning the area with a UV source, the locations of buried explosives are revealed. A mild irradiation of the area with electromagnetic energy in order to increase vapor concentration in the vicinity of buried explosives was also suggested.

Burlage did not describe the promoter used for inducing the reporter gene, nor did he specify the host organism used (both *Pseudomonas putida* and *Bacillus subtilis* are mentioned). Although partially successful preliminary field tests were reported (MacDonald et al., 2003), we are not aware of any reports describing further developments of this system.

One of the difficulties reported in the concept described above is that direct dispersion of the bacteria on dry soils resulted in the immediate absorbance of the bacteria to the soil, leading to rapid signal loss (Burlage, 2003). One manner by which this could be at least partially circumvented is by encapsulating the bacteria in a water and nutrient-retaining polymeric matrix (Bjerketorp et al., 2006). Several polymers have been reported over the years to be suitable for such purposes including alginate (Zohar-Perez et al., 2002), agar–agar (Kar et al., 2009), or gelatin (de las Heras and de Lorenzo, 2011). For mechanical dispersion of such immobilized bacteria over large areas, it is likely that encapsulation will need to be in a micro-bead format.

**FIGURE 2:**
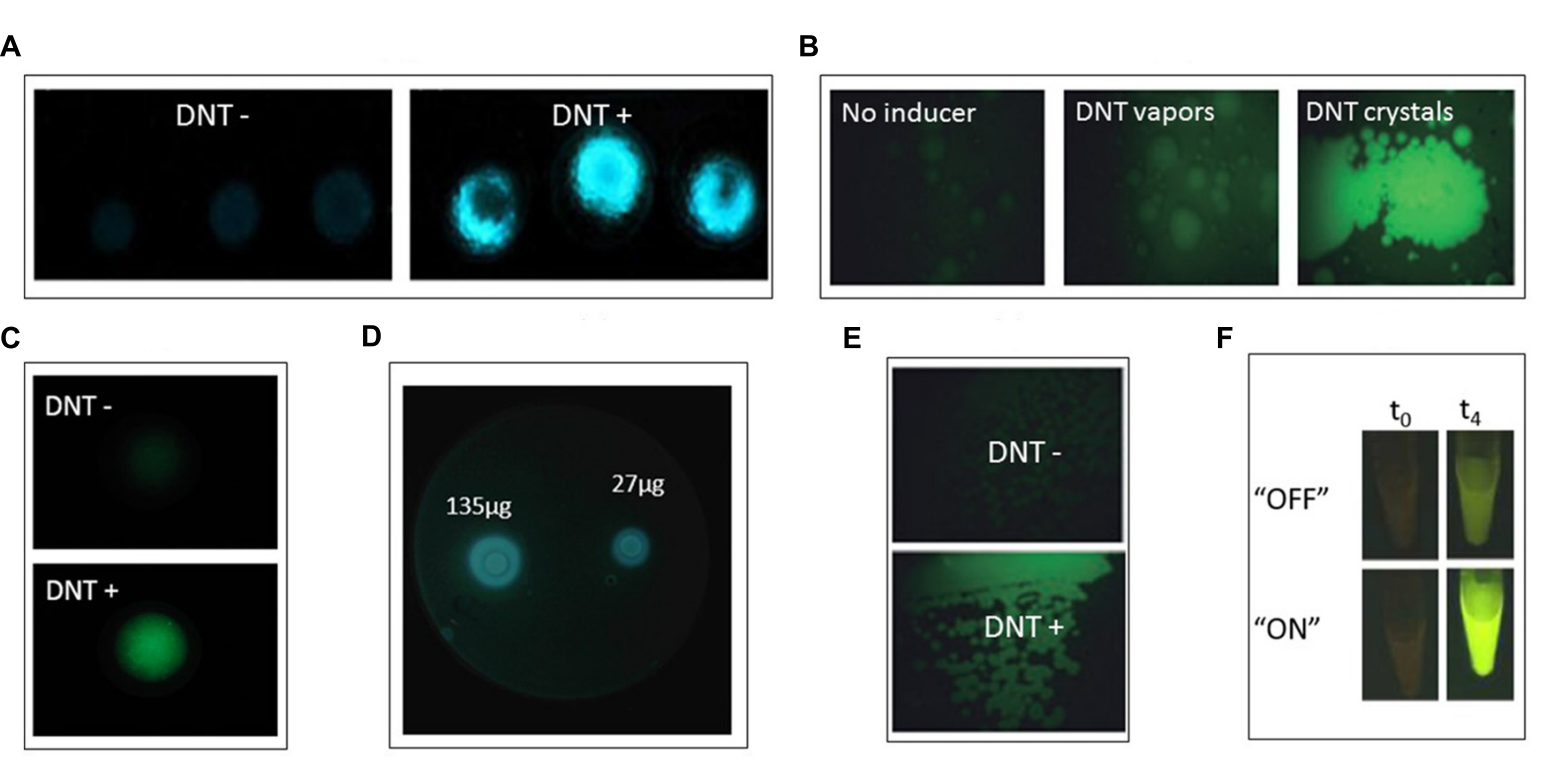
**(A,B)** *yqjF*-based bioreporter response when immobilized in agar and exposed to DNT buried in soil; **(A)** bioluminescent *lux*-based reporter; **(B)** fluorescent *gfp*-based reportet (from Yagur-Kroll et al., 2014, by permission; copyright (2013) Springer-Verlag Berlin Heidelberg). **(C)** *yqjF*-based bioluminescent bioreporter response when spread on LB agar plates and exposed to disks soaked with varying amounts of DNT (Yagur-Kroll et al., unpublished). **(D)** *xylR5*-based bioreporter response to DNT vapors and DNT crystals (from Garmendia et al., 2008, by permission). **(E)** *xylR5*-based bioreporter fluorescent response when spread on LB agar plates supplemented with DNT (from Garmendia et al., 2008, by permission; copyright (2008) Society for Applied Microbiology and Blackwell Publishing Ltd.). **(F)** Riboswitch-based bioreporer response to DNT when riboswitch is in “ON” or “OFF” mode at time zero and after 4 h of exposure. Reprinted (adapted) with permission from Davidson et al. (2012). Copyright (2012) American Chemical Society.

Yagur-Kroll et al. (2014) described an *Escherichia coli* bioreporter for the detection of TNT, DNT, and DNB (**Figure 2A-C**). A library containing approximately 2,000 *E. coli* clones, each bearing a plasmid with the *GFPmut2* gene fused to a different gene promoter, was screened for response to 2,4-DNT. Two gene promoters that exhibited the strongest response, *yqjF* (encoding a predicted quinol oxidase subunit) and *ybiJ* (encoding a protein of unknown function), were cloned into a low copy plasmid expressing the *P. luminescens luxCDABE* genes. These strains displayed a distinct dose-dependent response to 2,4-DNT, TNT, and 1,3-DNB. Interestingly, the reporter strain harboring the *yqjF* gene promoter as the sensing element was not induced directly by 2,4-DNT or TNT, but rather by metabolites of these compounds.

In an attempt to improve the capabilities of the constructed reporter in terms of detection threshold, signal intensity and time of detection, Yagur-Kroll et al. (2015) employed a “directed evolution” approach, involving four rounds of random mutagenesis of the *yqjF* promoter region by error prone PCR. The process yielded a variant that exhibited an over 3000-fold increase in luminescent signal intensity in the presence of 2,4-DNT, a 50-fold increase in the response ratio, a 75% reduction in the detection threshold and a response time that was cut down to half. An analysis of the point mutations accumulated in the course of this process indicated that the major contributors to these effects were manipulations of the -35 element of the *yqjF* gene promoter.

Tan et al. (2015) applied a similar approach to construct a bioreporter for the detection of nitroaromatic explosives, but instead of using a single gene promoter as the sensing element, five promoters found to respond to TNT, DNT, and DNB were fused to GFP. With this design, a detection threshold of 20.9 μM for TNT was obtained.

A non-specific use of a bacterial reporter was described by Frische (2002), who assessed the suitability of the luminescent bacterium *Aliivibrio fischeri* (formerly *Vibrio fischeri*) as a tool to detect the toxicity of TNT and its metabolites in soil samples. By combining chemical analysis that determined the concentrations of TNT and its degradation products, and the *A. fischeri* “lights off” assay for assessing the toxic effects, Frische (2002) was able to determine whether TNT is the main toxicant in a soil sample.

A principle common to most reports describing the design and construction of bacterial bioreporters is that the selected sensing element is nearly always based on a gene promoter activated in the presence of the target compounds. When such a gene has not been identified, an alternative approach may be to genetically manipulate a gene encoding a protein that binds similar molecules, thus modifying its binding site to recognize a new target. This has been the strategy employed by Galvão and De Lorenzo (2006), who made use of the *P. putida* XylR protein, which contains a domain that interacts directly with toluene and controls the activity of the σ54-dependent *Pu* promoter of the TOL plasmid for biodegradation of toluene and xylene (**Figure 2D-E**). By changing this domain through shuﬄing of its DNA sequence with a similar domain of the homologous protein DmpR, a XylR mutant that selectively binds 2,4-DNT was found (Garmendia et al., 2001). This mutant was expressed in a plasmid and inserted into *P. putida* strain Pu:GFP, in which GFP is expressed under the control of the Pu promoter. This bioreporter was induced by 2,4-DNT in a model soil setup (**Figure 2D**). The fact that *P. putida* is a soil bacterium renders it favorable as a bioreporter host for landmines detection (Garmendia et al., 2008).

A different approach for modifying regulatory protein specificity so that it responds to compounds it initially had little or no affinity to was attempted by Looger et al. (2003), who used a computational method to redesign ligand-binding-site specificity in proteins. The algorithm used high resolution three-dimensional structures to identify amino acid sequences predicted to form a complimentary surface between the protein and the target ligand. This algorithm was employed to engineer a new binding site for TNT that replaced the wild-type binding site for a ribose binding protein, a member of the *E. coli* periplasmic binding protein superfamily. It was claimed that the redesigned protein acquired an affinity to TNT, and that the receptors could distinguish the absence of a single nitro or methyl group. When integrated into a synthetic two-component signal transduction pathway in *E. coli*, the system was reported to have been activated by TNT. This report, however, was contested by Reimer et al. (2014) who demonstrated that there was no binding of TNT to the purified protein, nor was there an induction of the signal transduction pathway.

Two existing transcriptional factors in bacteria that were found to harbor the ability to bind 2,4-DNT were DntR from *Burkholderia* sp. (Suen and Spain, 1993) and NtdR in *Acidovorax* sp. strain JS42 (Lessner et al., 2003). Both are LysR-type transcription regulators and their amino acid sequence is 97% identical. NtdR, which activates the expression of genes involved in 2-nitrotoluene degradation, responds to 2,4-DNT and several other nitroaromatic compounds (Ju et al., 2009). Since NtdR has a broader range of ligands than DntR, it was selected as a starting point for a directed evolution process, initially by mutagenic PCR and then by recombination of the mutated sequences using staggered PCR. A mutant was obtained which displayed a 2,4-DNT detection limit of 10 μM, a 25-fold improvement compared with the WT strain. A combination of two mutations affecting the ligand-protein conformation was instrumental in the improvement of the binding (Lönneborg et al., 2012).

Employing a different approach, Kim et al. (2008) proposed to make use of the *E. coli* flagellar motor sensitivity to the presence of nitrate and nitrite; A glass-tethered *E. coli* strain KAF95, which carries a *cheY* gene deletion and is thus capable only of counterclockwise flagellar rotation, almost instantaneously stops its rotation in the presence of nitrate and nitrite. Flagellar motion was monitored by a microscope equipped with a CCD camera, and detection limits were reported to be 2.5 mM and 12 mM for nitrate and nitrite, respectively. However, responses to nitroaromatics were not demonstrated, and the obvious problem of false positives due to natural nitrates was not addressed.

Detection of DNT by a bioreporter that was designed outside the promoter-reporter fusion concept was reported by Davidson et al. (2012), based on riboswitch engineering (**Figures 2D-E**). A riboswitch is an element found in the 5′ untranslated region of some RNAs that has the ability to bind specific target molecules. The binding changes the secondary structure of the RNA, thus leading to a change in gene and protein expression. The riboswitch is composed of two components: a binding component (an aptamer) and an expression platform. A TNT binding aptamer (Ehrentreich-Förster et al., 2008), coupled with a PCR-generated expression platform placed upstream of the gene encoding tobacco etch virus protease, comprised the sensing component of the biosensor. Upon binding of the target DNT to the aptamer the expressed protease cleaved the binding between a GFP molecule and a yellow fluorescence protein that – when bound – quenched its fluorescence. The ensuing green fluorescence, when the system was expressed in *E. coli*, allowed detection of 0.5 mM DNT.

### Yeast

Although the study of yeast strains as bioreporters for environmental contaminants is quite extensive (Belkin, 2003; Sanseverino et al., 2005; Xu et al., 2013) and degradation mechanisms of certain explosives by yeast strains have been characterized (Zaripov et al., 2002), reports of yeasts as explosives bioreporters are very limited. One notable attempt was reported by Radhika et al. (2007), who inserted the primary components of the rat olfactory system to the yeast *Saccharomyces cerevisiae*.

The olfactory receptors (ORs) in mammalian organisms are activated by very specific odorants, resulting in the stimulation of the G protein G_olf_ (Jones and Reed, 1989). This eventually leads to the synthesis of cyclic AMP (cAMP) which stimulates a Ca^2+^ channel and increases Na^+^ and Ca^2+^ influx, thus creating an action potential which eventually reaches the central nervous system and is translated to an odor sensation. Screening of various ORs and their coupled G-proteins (GPCRs) for response toward 2,4-DNT resulted in the identification of the Olfr226 OR.

In Radhika’s olfactory yeast strain WIF-1α, GFP expression is coupled to cAMP synthesis. Upon exposure to 2,4-DNT the Olfr225 OR and GPCR are stimulated, cAMP is synthesized, GFP is expressed and a dose-dependent fluorescent signal is produced. This bioreporter responded to 25 μM of 2,4-DNT; no detection limit was reported by the authors.

### Microalgae

Measuring chlorophyll *a* fluorescence can provide an indication of photosynthetic activity, which is directly linked to the organism’s well-being. Thus, when a photosynthetic cell is stressed, inhibition in chlorophyll *a* fluorescence may be observed (Schreiber et al., 1995). This response, however, is very unspecific; Sanders et al. (2001), for example, immobilized *Chlorella vulgaris* and showed that by measuring the relative fluorescence of the cells it is possible to detect airborne chemical warfare agents. To make use of this phenomenon in a more specific manner, Altamirano et al. (2004) compared the inhibition of chlorophyll *a* fluorescence in the WT and a TNT-resistant strain of the green microalga *Dictyosphaerium chlorelloides*. The reportedly TNT-specific difference in fluorescence between the two strains allowed the detection of TNT concentrations as low as 0.5 mg/L.

## Enhancing Bioreporters Performance

A common denominator to all reports of cell-based sensing of explosives is the fact that the detection thresholds displayed are not sufficiently low to detect the very low concentrations expected to exist above buried landmines and other explosives-containing military hardware. Equilibrium headspace concentrations of DNT and TNT vapors above TNT based landmines can be as low as 0.28 pg/mL and 0.077 pg/mL, respectively (Jenkins et al., 2001).

If the detection is based on a promoter element induced by an explosive metabolite rather than the explosive itself, as in the case described by Yagur-Kroll et al. (2014), understanding the degradation process can be critical for enhancing the bioreporter’s performance. Knockout mutations in selected downstream genes or overexpression of selected upstream genes, for example, may be used to increase accumulation or production, respectively, of the inducing metabolite.

Another possible approach is the use of mixed cultures; Páca et al. (2008) showed that a mixed microbial culture could degrade 2,4-DNT 15–20 fold faster than single strain cultures of the same consortia. Applying such consortia in which one strain is used as the reporting element, while others degrade the parent material, could result in higher concentrations of the inducing metabolite released to the environment, eventually permeating to the bioreporter cell and increasing the response.

Among the practical problems that may be involved in the use of existing bioreporters are the shelf life prior to field application and the expected difficulties in coping with environment factors such as extreme temperatures and water availability once in the field. One attractive manner by which these difficulties may be addressed is the use of alternative resistant hosts, including spore-forming ones, such as those reviewed by Knecht et al. (2011). Another very important issue that has not been sufficiently addressed in many of the publications reviewed herein is the reporters’ specificity. For a viable field application it will be essential that the bioreporters used will respond only to a very limited range of target chemicals and their degradation products, as has been demonstrated by Yagur-Kroll et al. (2014). This specificity will be mostly dictated by the molecular elements selected as the sensing entities in the reporter construction, the manipulation of which should ensure minimal occurrence of false positives without compromising detection sensitivity.

## Conclusion and Outlook

The last two decades have witnessed significant progress in design and construction of bioreporters for the detection and monitoring of diverse environmental contaminants. As highlighted in the present review, this was not accompanied by parallel advances in the development cell-based sensors for the detection of explosives, a field which has received only a limited attention. While each of the bacterial reporters described to date may be a promising candidate for a future scheme for the detection of buried explosives, significant progress has yet to be made before such a scheme may be deemed practical.

The use of bioreporters for the detection of explosives, or other pollutants with environmental significance, is not without limitations. First and foremost, significant enhancement of currently reported detection sensitivity needs to be obtained. At the moment, detection thresholds appear to be inferior to analytical detection methods such as GC/MS or LC/MS, able to detect concentrations in the nM range, while most bioreporters do not perform well below 0.1 μM (Van der Meer and Jaspers, 2004). Some of the potential research avenues by which this objective may be achieved have been outlined above; other directions to be pursued include the use of additional microbial hosts, as well as additional basic studies of the molecular mechanisms and biochemical pathways by which microbes metabolize explosives’ molecules or respond to their presence.

Another point that should be considered is the nature of the steps that need to be taken to ensure that the released bacteria survive in the field for periods that are sufficiently long to allow them to respond to their inducers and generate a readable signal, but also prevent their subsequent proliferation. The latter issue is essential in view of the genetically engineered nature of the bioreporters, even if by themselves they do not constitute any environmental or human safety risk. Relevant regulations will need to be adhered to, and public opinion issues considered. Possible precaution steps that may be taken include an engineered auxotrophy to nutrients unavailable in the environment, or the introduction of a “suicide circuit” that will not allow environmental survival (García and Díaz, 2014).

It should also be remembered that in many cases bacterial bioreporters may display detection thresholds that are inferior compared to chemical analysis. Their main advantage may lay in their ability, once released in the field, to act as independent agents and generate a dose-dependent signal that may be monitored from a distance. Thus, in parallel to the continuous development of better microbial sensors, attention should be devoted to the engineering of the hardware required for the remote detection of their optical signals and for pinpointing their activity hotspots.

## Conflict of Interest Statement

The authors declare that the research was conducted in the absence of any commercial or financial relationships that could be construed as a potential conflict of interest.
